# Framingham Coronary Heart Disease Risk Score Can be Predicted from Structural Brain Images in Elderly Subjects

**DOI:** 10.3389/fnagi.2014.00300

**Published:** 2014-12-01

**Authors:** Jane Maryam Rondina, Paula Squarzoni, Fabio Luis Souza-Duran, Jaqueline Hatsuko Tamashiro-Duran, Marcia Scazufca, Paulo Rossi Menezes, Homero Vallada, Paulo A. Lotufo, Tania Correa de Toledo Ferraz Alves, Geraldo Busatto Filho

**Affiliations:** ^1^Laboratory of Psychiatric Neuroimaging (LIM 21), Department of Psychiatry, Faculty of Medicine, University of São Paulo, São Paulo, Brazil; ^2^Centre for Computational Statistics and Machine Learning, Department of Computer Science, University College London, London, UK; ^3^Núcleo de Apoio à Pesquisa em Neurociência Aplicada (NAPNA), University of São Paulo, São Paulo, Brazil; ^4^Department and Institute of Psychiatry, University of São Paulo, São Paulo, Brazil; ^5^Department of Preventive Medicine, University of São Paulo, São Paulo, Brazil; ^6^Center for Clinical and Epidemiologic Research, University of São Paulo, São Paulo, Brazil

**Keywords:** Framingham score, cardiovascular risk factors, magnetic resonance imaging, pattern recognition, multivariate analysis

## Abstract

Recent literature has presented evidence that cardiovascular risk factors (CVRF) play an important role on cognitive performance in elderly individuals, both those who are asymptomatic and those who suffer from symptoms of neurodegenerative disorders. Findings from studies applying neuroimaging methods have increasingly reinforced such notion. Studies addressing the impact of CVRF on brain anatomy changes have gained increasing importance, as recent papers have reported gray matter loss predominantly in regions traditionally affected in Alzheimer’s disease (AD) and vascular dementia in the presence of a high degree of cardiovascular risk. In the present paper, we explore the association between CVRF and brain changes using pattern recognition techniques applied to structural MRI and the Framingham score (a composite measure of cardiovascular risk largely used in epidemiological studies) in a sample of healthy elderly individuals. We aim to answer the following questions: is it possible to decode (i.e., to learn information regarding cardiovascular risk from structural brain images) enabling individual predictions? Among clinical measures comprising the Framingham score, are there particular risk factors that stand as more predictable from patterns of brain changes? Our main findings are threefold: (i) we verified that structural changes in spatially distributed patterns in the brain enable statistically significant prediction of Framingham scores. This result is still significant when controlling for the presence of the *APOE 4* allele (an important genetic risk factor for both AD and cardiovascular disease). (ii) When considering each risk factor singly, we found different levels of correlation between real and predicted factors; however, single factors were not significantly predictable from brain images when considering APOE4 allele presence as covariate. (iii) We found important gender differences, and the possible causes of that finding are discussed.

## Introduction

Cardiovascular risk factors (CVRF) such as hypertension, diabetes, dyslipidemia, and smoking, are highly prevalent in the elderly population and have significant impact on cognitive performance. In the last years, effects of CVRF on brain morphology have been widely investigated using neuroimaging resources. A number of morphometric studies using magnetic resonance imaging (MRI) (Korf et al., 2007; Almeida et al., 2008; Chen et al., 2012; Beauchet et al., 2013) have found decreased gray matter (GM) volumes in direct proportion to the degree of cardiovascular risk in otherwise healthy elderly individuals. In addition, studies using functional imaging methods such as positron emission tomography (PET) or functional MRI (fMRI) (Minoshima et al., 1997; Chetelat et al., 2003; Jagust, 2006; Kawachi et al., 2006; Mosconi et al., 2007; Marchand et al., 2011) have reported brain activity deficits also correlated with cardiovascular risk. These neuroimaging alterations often involve brain regions known to be implicated in the pathophysiology of Alzheimer’s disease (AD), such as the hippocampal region, posterior cingulate gyrus, temporal, and parietal cortices (Korf et al., 2004; Seshadri et al., 2004; Riello et al., 2005; Chen et al., 2006; Viswanathan et al., 2009; Neufang et al., 2011, 2013; Rasgon et al., 2011). Thus, such findings have provided critical support to the notion that the degree of cardiovascular risk may be a key influence in the development and course of AD.

Several studies applying neuroimaging to investigate the impact of cardiovascular risk on the brain have one important limitation, namely to consider each factor singly. As CVRF are widely known to seldom appear in isolation (Wilson et al., 1998; Jeerakathil et al., 2004; Seshadri, 2006; Razay et al., 2007), the influence of combined CVRF in imaging studies is likely to improve sensitivity to such investigations. Recently, our group examined GM changes in structural MRI data from elderly subjects with different degrees of cardiovascular risk (de Toledo Ferraz Alves et al., 2011) using the Framingham Coronary Heart Disease Risk [FCHDR (Grundy et al., 1998; Wilson et al., 1998)] score, a composite index comprising five clinical factors (age, blood pressure, diabetes mellitus, smoking status, and cholesterol levels). Given recent findings regarding cardiovascular risk effects on cognitive performance (Mosconi, 2005; Obisesan et al., 2008; Fitzpatrick et al., 2009; Purandare, 2009; Scarmeas et al., 2009; Buchman et al., 2012), the FCHDR score has presented excellent potential for investigations involving brain imaging (Elias et al., 2003; Jeerakathil et al., 2004; Massaro et al., 2004; Seshadri et al., 2004; DeCarli et al., 2005; Romero et al., 2009).

For the study by de Toledo Ferraz Alves et al. (2011), dementia-free individuals aged from 66 to 75 years were recruited from the database “São Paulo Aging and Health Study” (SPAH) (Scazufca and Seabra, 2008; Scazufca et al., 2008) and divided into three subgroups (high-risk, medium-risk, and low-risk) according to their FCHDR scores and gender (Table 1). Analyses of covariance were performed to investigate the presence of mean regional GM volume differences between the subgroups, followed by *post hoc t*-tests.

**Table 1:** **Division of subjects in risk groups according to FCHDR in males and females**.

Group	Male	Female
Low risk	Score ≤5	Score ≤9
Medium risk	5 < Score ≤8	9 < Score ≤14
High risk	Score >8	Score >14

Using voxel-based morphometry (VBM) (Ashburner and Friston, 2000), the authors showed that the presence of CVRF was associated with mean regional GM loss involving the lateral temporal cortices bilaterally. They also observed a negative correlation between FCHDR scores and regional GM distribution in the lateral parietal cortex. Both areas are considered critical to pathophysiology of AD (Garrido et al., 2002; Mosconi et al., 2007; Yakushev et al., 2009; Ystad et al., 2009).

Several studies in this area do not take into account information regarding the major genetic risk factor for AD – the epsilon 4 (ε4) variant of the gene encoding the apolipoprotein E (*APOE* allele 4, *APOE* *4, or *APOE* 4) (Reiman, 2007; Cherbuin et al., 2008). This is a limitation since the presence of the APOE-4 allele carrier is also a risk factor for cardiovascular diseases and may be associated with variations in brain structure and functioning, even in non-elderly adults (Kivipelto et al., 2008). In our previous VBM investigation (de Toledo Ferraz Alves et al., 2011), regional GM differences across groups retained significance when considering the *APOE* 4 allele as a covariate of interest. This finding brought additional support to the hypothesis of a direct effect of CVRF on the brain, and consequently, a possible non-genetically determined influence on the development of AD-like brain abnormalities caused by microvascular-related changes in the general population.

The results of our previous investigation are, however, likely to have been influenced by common limitations in brain imaging studies. These include the increased chance of type I errors due to the large number of statistical comparisons across the entire brain, as well as the univariate, voxel-by-voxel nature of conventional VBM approaches that force investigators to address brain abnormalities pertaining to each brain compartment (GM, white matter, and cerebrospinal fluid space) separately. In the present study, we aimed to address these limitations using multivariate analysis. This approach takes into account covariance relations across brain regions/voxels, rather than a voxel-by-voxel comparison. The covariance approach can present greater statistical power in comparison with univariate techniques, as corrections for voxel-wise multiple comparisons are not necessary. Additionally, as voxels from the whole brain are modeled together, their results can be interpreted as a signature of neural networks.

Another critical limitation of conventional VBM studies is related to their restriction to mean group comparisons, with no information affordable for individual subjects. Analyses of individual subjects are feasible using contemporary pattern recognition (PR) methods. These methods are relevant as they provide indices of diagnostic accuracy, and these can be potentially useful to measure individual risks of favorable outcomes. PR methods for classification have been increasingly applied in the field of neurodegenerative dementias. Classifiers using morphometric MRI data have presented high indices of diagnostic accuracy that reinforce the diagnosis of AD, the commonest form of dementia (Vemuri et al., 2008; Magnin et al., 2009; Plant et al., 2010; Dai et al., 2012; Liu et al., 2012). These techniques have also afforded promising results in studies related to MCI – cognitive decline not severe enough to fulfill the criteria for established dementia (Misra et al., 2009; Aksu et al., 2011; Chincarini et al., 2011; Cui et al., 2011; Davatzikos et al., 2011; Zhang and Shen, 2012). However, the latter is still an open area with intense investigations, as predicting whether individuals who are at increased risk convert to AD is more challenging than classifying AD versus control individuals.

Given that CVRF are nowadays recognized as important risk factors for AD, it is relevant to investigate whether PR techniques are capable of discriminating individuals with variable degrees of cardiovascular risk based on MRI data. Another important approach in the present work is to apply a multivariate regression method in order to evaluate whether it is possible to predict the FCHDR score individually. Additionally, we explored single CVRF to investigate if any of those single factors carry enough information to predict brain changes in these individuals when a pattern classification approach is applied.

## Materials and Methods

### Study sample

Subjects used in the analysis were described in a previous publication (de Toledo Ferraz Alves et al., 2011). All subjects were recruited from a population sample addressed in an epidemiological research program, the SPAH study (Scazufca et al., 2008).

**Figure 1:**
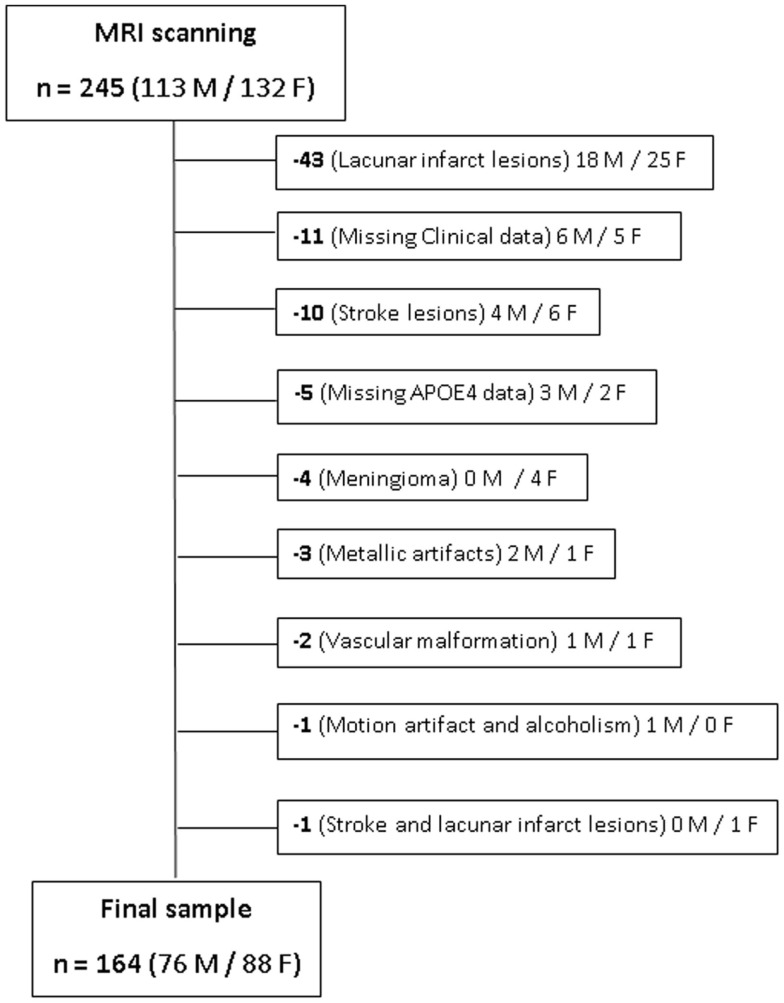
**Representation of sample selection and exclusion cases**. The number and gender (M/F) of each subject excluded from the original sample are presented along with the respective reasons for exclusion.

The inclusion criterion was elderly individuals aged between 66 and 75 years. Exclusion criteria were as follows: (a) presence of dementia or minor cognitive impairment; history of stroke, epilepsy, brain trauma, transitory ischemic events and other neuropsychiatric disorders; and contra-indications for MRI scanning. After MRI acquisition, additional individuals were excluded due to different reasons, as artifacts, presence of silent gross brain lesions identified on the images and individuals for which one or more of the variables needed to generate the FCHDR score were missing. Figure 1 presents this information, where the reasons for exclusions, as well as the number of subjects excluded and their gender distribution were detailed. The study was approved by local ethics committees, and all subjects gave informed written consent.

Table 2 presents demographic and clinical characteristics for the final sample of 164 subjects whose MRI scans were used in the present analyses. For classification, these 164 subjects were divided into three groups: low, medium, and high cardiovascular risk according to their FCHDR score, using the criteria in Table 1 in order to accommodate gender differences.

**Table 2:** **Demographic and clinical characteristics of the subjects in the sample analyzed**.

	Low risk (*n* = 42)	Medium risk (*n* = 65)	High risk (*n* = 57)	Statistical test	*p*-Value
Mean age (±SD) in years	70.19 ± 2.26	70.17 ± 2.31	70.63 ± 2.45	ANOVA	0.503
Male/female	9/33	23/42	44/17	χ^2^	<0.001
Mean years of education (±SD) in years	4.59 ± 3.33	5.14 ± 4.02	3.86 ± 3.31	ANOVA	0.152
*APOE_*4 allele *n* (%)	9 (21.43%)	17 (26.15%)	10 (17.54%)	χ^2^	0.516

Genomic DNA was extracted from EDTA-anticoagulated venous blood using a standard salting-out method and its concentration was determined by spectrophotometric measurements. The single nucleotide polymorphisms (SNPs) rs429358 and rs7412 that determine the three APOE isoforms (APOE ε2, APOE ε3, and APOE ε4) were genotyped in two different laboratories. Initially, the genotyping was performed at the laboratory of Genetics and Pharmacogenetics Program at the Institute of Psychiatry, University of Sao Paulo. Then, for quality control, the genotyping was also performed under contract by Prevention Genetics (http://www.preventiongenetics.org). The degree of result discrepancy between the two labs was below 2%.

We report herein the results obtained in the analysis carried out by Prevention Genetics. Positive APOE4 status was considered by the presence of at least one allele ε4 in the genotype (i.e., both heterozygous and homozygous carriers included). In addition, a Hardy–Weinberg equilibrium test was performed and no statistically significant deviation between the loci was observed. In the analyses for the current study, as in the previous investigations by de Toledo Ferraz Alves et al. (2011), we opted for taking into account only the effect of the *APOE* allele 4. This was due to the fact that the number of subjects was not large enough to address the complexity of the combined effect of *APOE* alleles 2 and 4.

### Brain images: Acquisition and pre-processing

Imaging data were acquired using a 1.5-T GE Sigma scanner (General Electric, Milwaukee WI, USA), according to the following T1-SPGR protocol: (a) a dual-spin-echo sequence of 120 transaxial slices across the entire brain (axial PD/T2); (b) a T2-weighted fast spin-echo transaxial sequence with 88 slices; and (c) a three-dimension gradient echo (Spoiled Gradient Recalled Acquisition–SPGR) sequence of 124 slices with TR/TE of 21.7/5.2 msec, flip angle of 20°, 220 mm field of view (FOV), 1.5 mm slice thickness, number of measures (NEX) of 01, 256 × 192 matrix. All images were visually inspected by an experienced radiologist with the purpose of identifying artifacts and presence of silent gross brain lesions.

After quality inspection, T1 images were pre-processed using DARTEL (Ashburner, 2007). Initially, segmentation was performed in order to generate gray and white matter images of each subject aligned via a rigid-body transformation. After segmentation, templates were created in order to align gray and white matters simultaneously across the images. Finally, images were spatially normalized to MNI space and smoothed using a Gaussian 8 mm FWHM. For this study, we used *swc1* images (non-modulated, normalized, and smoothed images) where each voxel intensity represents the probability of containing GM. Before the analysis, we applied a threshold of 0.15 to the GM probability maps (i.e., voxels with less than 15% of probability of containing GM were discarded in all images).

### Framingham score and other clinical assessment schedules

The FCHDR is a measure devised to synthesize the combination of different CVRF in order to predict a major vascular-related event (Grundy et al., 1998; Wilson et al., 1998). These CVRF include: age, blood pressure, diabetes mellitus, smoking status, and cholesterol levels (Korf et al., 2004; Lopez et al., 2008; Fitzpatrick et al., 2009; Zivadinov et al., 2009; de Toledo Ferraz Alves et al., 2010; Rasgon et al., 2011).

The clinical tools used to assess the individuals recruited for the study included: measurements of fasting blood glucose levels, with the presence of diabetes mellitus was defined by ≥126 mg/dl and/or current use of insulin or hypoglycemic oral drug treatment; levels of total cholesterol, and high density lipoprotein (HDL), obtained using the cholesterol-oxidase method; and measures of blood pressure, obtained in three measurements using an OMRON digital sphygmomanometer; model HEM-712-C. Individuals with high blood pressure were under antihypertensive treatment. For the calculation of the arterial pressure value, the first measurement was discarded, and the arithmetic mean of the second and third measurements was calculated. Data regarding smoking habits were provided by participant’s answers. More details regarding clinical measurements can be obtained in de Toledo Ferraz Alves et al. (2010) and Tamashiro-Duran et al. (2013).

### Pattern recognition

In the present paper, we used machine learning techniques, a branch of artificial intelligence concerned with development of algorithms able to evolve based on empirical data. Machine learning algorithms use examples (data) to capture characteristics of interest from their underlying probability distribution (unknown *a priori*). Data can be represented as observations (*examples*) that contain relations among variables (*features*). In structural neuroimaging, each example is usually a scan (brain volume) from a particular subject and features usually correspond to voxels composing the images (or voxels composing a region of interest anatomically defined). One of the major focuses of machine learning relies on automatic recognizing complex patterns and making decisions based on data (PR). Hence the learner algorithm must generalize from the given examples, as well as to be able to produce an output for new cases not used in the training set.

#### Classification

One of the most common objectives of PR algorithms in machine learning is classification, which attempts to assign each input value to one of a given set of classes. In neuroimaging, classification approaches have become increasingly popular in clinical research due to their ability to produce individual predictions. In the context of our problem, we hypothesize that the examples (brain scans) of subjects belonging to the low-risk group can be discriminated from the high-risk group with higher accuracy than in classifications involving the medium-risk group.

In general, classification methods work as follows: given a set of training examples, each one known to belong to a specific category (class), the training algorithm learns a function based on the values of each variable in the training set. The decision function learnt is used to classify a new example (i.e., to predict the category to which it belongs) based on to the values of their variables.

In neuroimaging, variables (features) composing the examples usually correspond to voxels derived from a brain scan. Thus, usually each example comprises thousands of features. Although it is not possible to visualize more than three dimensions, mathematically it is possible to conceive a hyperspace where each feature is mapped into a different dimension. In this paper, we applied support-vector machine (SVM) (Boser et al., 1992; Cortes and Vapnik, 1995). The problem of obtaining a decision function in SVM consists in finding a hyperplane (a plane in a hyperspace), which has the largest margin between the closest examples across classes (called support vectors).

##### Relation between classification projections and FCHDR scores

In the current paper, we performed three binary classifications combining each of the three groups (two by two) (medium versus low risk, high versus medium risk, and high versus low risk). Additionally, as a binary classification may not be enough to define precise predictions in some clinical applications (Sato et al., 2008), we also performed a more exploratory analysis in order to investigate the effect of the degree of cardiovascular risk (treated as a continuous measure) in classifying the groups (categorical information).

According to Sato et al. (2008), in many clinical applications, a continuous index can be more informative than categorical labels, as the distance from the classification boundary reflects prediction reliability. These authors mention the example of AD studies in which two groups of subjects (healthy subjects and patients) are considered. In this case, it would be reasonable to expect that the cognitive effects may vary across subjects; depending on the disease stage (degree of dementia), similar levels of cognitive performance might be present in some of the healthy subjects. In these cases, a continuous variable representing the mental condition of a subject might be considered.

Sato et al. (2008) take into account the fact that there are intermediate observations (a group transition), so that categorical observations might be localized in a continuum between two groups. To illustrate this idea, they apply the maximum uncertainty linear discriminant analysis approach (MLDA) hyperplane navigation (Thomaz et al., 2006), specifying a procedure to project multi-dimensional observations from different groups (categorical label) into a continuum space, obtaining a score to measure the distance of new observations to each group. This score is afterward correlated to the real continual values (in their case, age in a group of healthy subjects performing a motor task in fMRI).

Considering the idea explained above, we performed in the present study a correlation analysis of the projection of the samples in the hyperplane with their FCHDR scores. The rationale is to evaluate the link between brain image patterns and cardiovascular risk information, based on the assumption that, in general, the more extreme the risk, the further the sample would be distant from the decision boundary.

#### Regression

**Figure 2:**
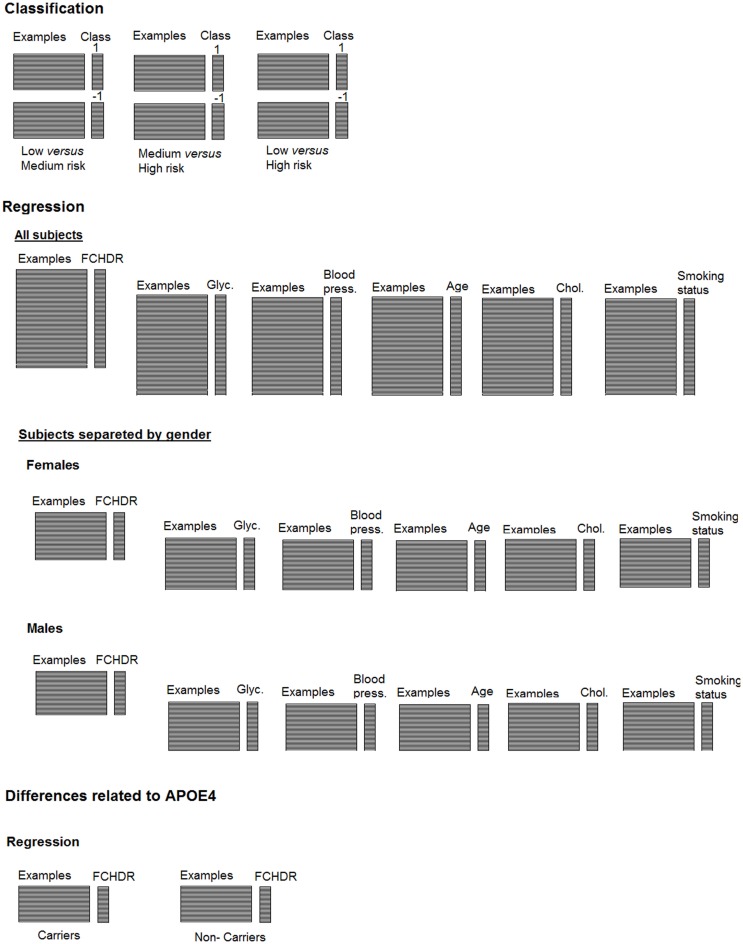
**Representation of classification and regression analysis described along the paper**.

For classification analysis, subjects were categorized in three groups according to their FCHDR scores. Although classification is the most common PR approach used in neuroimaging, machine learning techniques can also be applied to non-categorical data through regression methods. This approach has great potential to be applied in clinical researches involving continuous data (e.g. population or physiological data, behavioral tests results, etc). In this paper, we used support-vector regression [SVR (Drucker et al., 1997; Smola and Schölkopf, 1998)], an algorithm similar to SVM for classification, nevertheless applying different parameterization. Instead of predicting a category to which an example belongs, the objective here is to predict the clinical measure itself. Figure 2 shows a representation summarizing both the classification and regression analyses presented in this paper.

The predictors for the analysis of single risk factors were normalized (i.e., the mean value was subtracted from the original values and results were divided by the SD). The normalization enabled comparison of mean square errors (MSE) among different single risk factors, as they were originally represented through different measure units. MSE measures the average of the square *errors* (i.e., the amount by which the value implied by the estimator differs from the quantity to be estimated). Both classification and regression methods were implemented using LIBSVM library^1^.

### Cross-validation framework

Cross-validation is a technique used to assess how the results of a statistical analysis will generalize to an independent data set. One round of cross-validation involves partitioning a data sample into disjoint subsets of examples, performing the analysis on one subset (the training set), and validating the analysis on the other subset (the validation set or testing set).

To reduce the variability, multiple rounds of cross-validation are performed using different partitions, and the validation results are averaged over the rounds. In the present study, we implemented a leave-one-out cross-validation (LOO-CV), which involves separating a single example from the original sample for validation while the remaining examples are used as the training dataset. This splitting is repeated in order that each example in the sample is used once as the validation data. After repeating this process leaving out all examples, the final accuracy was quantified as the average of accuracies obtained across all iterations. In classification, we left out one pair of examples (i.e., one subject from each group) in each round so that the analysis was balanced between the two groups. For regression analysis, one single subject was left out at each round, as the objective is not a classification between two groups.

### Statistical significance testing

We implemented permutation tests to evaluate whether the results were statistically significant. This approach is a non-parametric statistical test in which the distribution of the test under the null hypothesis is obtained by calculating many possible combinations under rearrangements of the labels (e.g., categories or clinical values) across the examples. It consists in randomly exchanging the labels associated to the examples and repeating the complete procedure a high number of times (e.g., 1000). As the correlations between examples and labels are destroyed due to the labels’ permutation, one expects the classification accuracy to be close to chance (around 50%).

The significance level is given by the number of times for which the accuracy obtained from analysis with permuted labels is higher than the accuracy obtained in the original analysis. The resulting number is divided by the number of repetitions to provide a proportion value. The same approach is applied to regression, considering the correlation between real, and predicted labels instead of accuracy.

## Results

### Classification of risk groups categorized according to FCHDR scores

As described in section “Study Sample,” we divided the individuals in three groups (low, medium, and high cardiovascular risk) according to their FCHDR scores. In order to deal with unbalance issues, we matched subjects by gender and age. This is important to keep generalization ability, particularly in a cross-validation approach. After matching subjects, we performed three binary classifications: low versus medium risk (84 subjects), medium versus high risk (74 subjects), and low versus high risk (44 subjects).

Given that the Framingham score takes into account age and gender, we tried an alternative approach, considering all subjects without matching. However, because of the different number of subjects in each group, randomness was applied in the cross-validation in order to select repeated subjects in the group with less subjects. Using this approach, average accuracy was slightly better, but there was a loss in generalization ability.

All the analyses were performed taking into account genetic information regarding *APOE-*4 allele carriers (data used as a covariate). In order to control for this effect, we applied a removal of confound effects on the kernels using a residual form matrix, as described in Chu et al. (2011).

Support-vector machine applied to classifications involving the medium-risk group (i.e., low versus medium risk and medium versus high risk) did not produce statistically significant results according to the permutation tests. Accuracies of both analyses were close to chance (Table 3), meaning that the classifiers were not able to discriminate these groups. However, it was possible to discriminate extreme groups (high versus low risk) with 75% accuracy. The sensitivity (i.e., the proportion of individuals from high-risk group correctly classified) was 82% and the specificity (i.e., the proportion of individuals from low-risk group correctly classified) was 68%. Permutation test showed this result was significant (*p* < 0.0001). Table 3 presents results from the three binary classifiers.

**Table 3:** **Results from SVM classification considering the three risk groups in binary combinations**.

Groups	Sensitivity (%)	Specificity (%)	Accuracy (%)	Statistical significance
Medium × low risk	38.10	59.52	48.81	≤0.6390
High × medium risk	62.16	43.24	52.70	≤0.3490
High × low risk	81.82	68.18	75	≤0.0001

Regarding the additional analysis described in sub-section “Relation between classification projections and FCHDR scores” above, the Pearson correlation value between FCHDR scores and SVM projections in the classification between high and low-risk groups was 0.788 (*p* ≤ 0.0001). The same correlation for the classifications involving the medium-risk group were 0.559 (*p* ≤ 0.0002) for high *versus* medium risk, and 0.609 (*p* ≤ 0.0001) for medium *versus* low risk.

**Figure 3:**
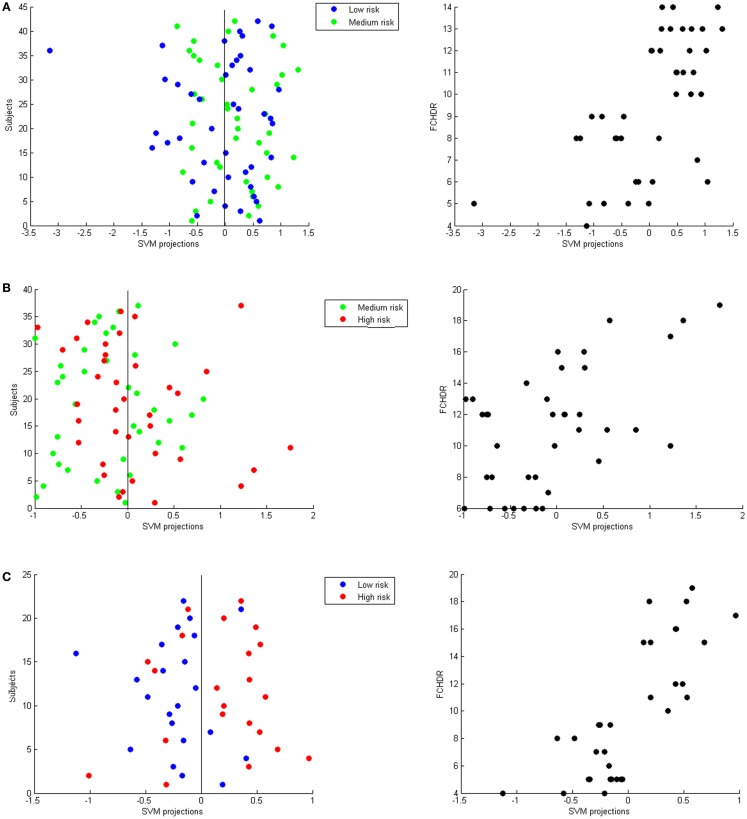
**Each panel presents SVM projections resulting from a binary classification: (A) Low versus medium risk; (B) Medium versus high risk; (C) Low versus high risk**. On the left-hand side in each panel, the plot shows the classification projection in colors representing the real class of each subject. As a convention, in each classification, the lower risk class was labeled with −1 and the higher class was labeled as 1. Thus, subjects with negative projections are classified as belonging to the lower risk class and subjects with positive projections are classified as belonging to the higher risk class. From these plots, it is possible to notice that the numbers of correct classifications in **(A,B)** are very close to chance. In **(C)**, it is possible to notice that most low-risk subjects have negative projections and most high-risk subjects have positive projections (which results in the accuracy of 75% described in Section “Classification of Risk Groups Categorized According to FCHDR Scores.” Nevertheless, for all classifications there was significant correlation between SVM projections and FCHDR for the subjects correctly classified (illustrated in the plots on the right-hand side in each panel).

Figure 3 presents three panels illustrating SVM classification projections and correlation of these projections with FCHDR for the three binary classifications – (A) low versus high risk; (B) low versus middle risk; and (C) middle versus high risk.

### Regression analysis

In this section, we present results of the regression analysis without categorization in risk groups, since we are interested in evaluating how accurately we can predict cardiovascular risk from brain patterns. For regression, we also used a cross-validation framework. However, only one subject is left out for test in each iteration (instead of one pair of subjects), as we are not comparing groups (classes) of individuals. We start using FCHDR scores as targets for regression. Next, in a more exploratory investigation we analyze risk factors that contribute to the FCHDR score computation (diabetes, blood pressure, age, cholesterol, and smoking status) in order to evaluate if there are factors that stand out as more predictable from the brain patterns. Additionally, we analyze data from male and female subjects separately, as differences in gender have been reported in related literature (Perneczky et al., 2007; Hsu et al., 2008; Ystad et al., 2009; Andersen et al., 2010; Takahashi et al., 2011; Yao et al., 2012; Hall et al., 2013).

#### All subjects

**Figure 4:**
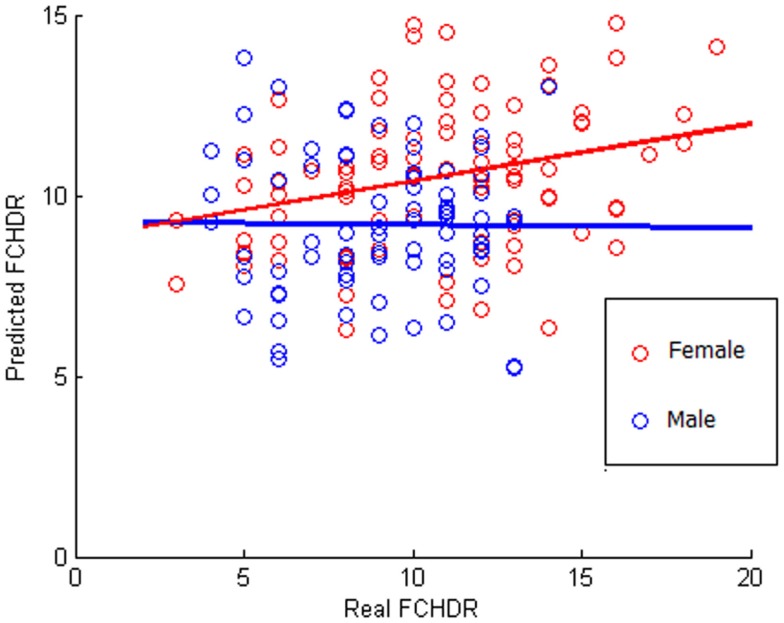
**Scatter plot of correlation between real and predicted FCHDR score codified in colors according to the gender**. Please notice that this analysis was performed considering all subjects (male and female). The colors code (red for female and blue for male) was used in order to provide a better visualization regarding the gender difference observed in the results.

The result of the correlation between real FCHDR and predicted values was 0.2481 (*p* < 0.001) with MSE 1.0564 (*p* < 0.001). In Figure 4, a scatter plot represents this correlation, where each subject is plotted with real FCHDR score placed in the *x*-axes and predicted score placed in the *y*-axes. Individuals were plotted in different colors according to gender. The color coding offers an initial insight regarding the existence of gender differences in relation to the association of brain patterns to CVRF.

Besides the application of SVR to learn relations between brain patterns and the combined measure of cardiovascular risks (FCHDR score), we also evaluated whether it is possible to predict single risk factors from relations learned from brain patterns. Results are presented in Table 4. No single factor presented significant results of the correlation between real and predicted variable. As regards dichotomic diabetes (derived from glycemia), we applied classification to evaluate whether is possible to discriminate diabetic and non-diabetic individuals. Like the other single risk factors, results for diabetes were not significant (classification accuracy = 55%, *p* = 0.22).

**Table 4:** **Regression with single risk factors**.

	*R*	*p*(*R*)	MSE	*p*(MSE)
FCHDR	0.2481	<0.001	1.0564	<0.001
Blood pressure	0.0753	0.215	1.1498	0.216
Age	0.0052	0.424	1.2408	0.517
Total cholesterol	−0.0212	0.529	1.3400	0.821
LDL cholesterol	−0.0475	0.642	1.3417	0.822
Smoking status	0.1628	0.057	1.0698	0.067

**R* is the correlation between real and predicted values and MSE is the mean squared error. The statistical significance (*p*) was estimated for both R and MSE*.

#### Gender differences

Figure 4 suggests that there is a potential difference in the ability of predicting FCHDR from brain patterns according to gender. As the FCHDR score was devised through different calculation for male and female individuals (see Study Sample), we also performed the regression analysis separately for male and female individuals. The correlation between real and predicted FCHDR scores in females was 0.4044 (*p* < 0.001). However, in males this correlation was not significant.

Table 5 presents results of analyses performed separately in females and males. For females, age and smoking status could be predicted from brain patterns. Blood pressure also stood out, but the statistical significance of its accuracy was slightly above the considered threshold of 0.05. For males, no single factor could be predicted (Table 5).

**Table 5:** **Regression with single risk factors**.

	*R*	*p*(*R*)	MSE	*p*(MSE)	*R*	*p*(*R*)	MSE	*p*(MSE)
FCHDR	0.4044	<0.001	0.8323	<0.001	−0.0057	0.424	1.1999	0.580
Blood pressure	0.2309	0.054	0.9685	0.052	−0.1945	0.850	1.3054	0.818
Age	0.2776	0.033	0.9379	0.035	0.1182	0.201	1.0267	0.136
Total cholesterol	−0.0478	0.540	1.1960	0.504	−0.0177	0.441	1.2608	0.702
LDL cholesterol	−0.1264	0.755	1.2691	0.725	−0.0376	0.480	1.2206	0.605
Smoking status	0.3480	0.008	0.8705	0.007	−0.0894	0.633	1.2826	0.780

**R* is the correlation between real and predicted values and MSE is the mean squared error. Statistical significance (*p*) was estimated for both R and MSE. These analyses were performed separately in females (left in the table) and males (right)*.

### *APOE* 4 allele differences

The presence of *APOE* 4 allele is associated with an increased risk not only for dementia but also for cardiovascular disease. Therefore, the APOE-4 allele carrier can be a confound factor for the analyses. It is important to evaluate if the correlation between real and predicted FCHDR scores remains significant if the APOE genotyping is included in the model as a covariate. In the analyses performed using all subjects (males and females), the results retained significance for FCHDR score (*r* = 0.1961, *p* = 0.007). Regarding the analysis performed only with female individuals, both FCHDR score and age retained significance when the genetic data was included as covariate in the model.

#### Regression analysis in *APOE*4 carriers and non-carriers separately

In our database, 36 individuals carry at least one *APOE*-4 allele. We selected a group consisting of 36 non-allele 4 carriers from the same database matching according to gender, age, and FCHDR score. We performed regression analysis separately for carriers and non-carriers using FCHDR as target. The correlation between real and predicted FCHDR was 0.4240 (*p* = 0.003) for carriers and 0.2937 (*p* = 0.041) for non-carriers.

**Figure 5:**
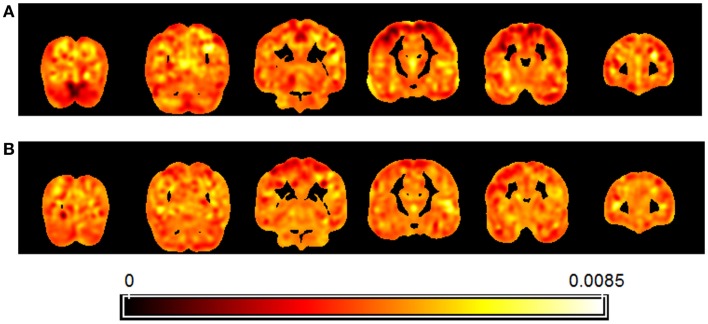
**Map showing the relative weight of each voxel in the multivariate regression (SVR) of images using FCHDR scores as targets**. Separate analysis was performed for APOE4 allele carriers **(A)** and non-carriers **(B)**.

Figure 5 presents a map of the relative weights of each voxel resulting from regression of brain patterns using FCHDR scores as targets in the analyses performed with carriers (Figure 5A) and non-carriers (Figure 5B).

Regarding the map, it is important to observe that due to the fact that the analysis is multivariate, the combination of all voxels is identified as a global spatial pattern by which the groups differ (i.e., the discriminating pattern). As all voxels contribute to the classification in some degree, it is not appropriate to make regional inferences based on single voxels or regions separately from the rest of the pattern.

## Discussion

Recent literature on neuroscience related to aging processes has shown increasing evidence of the impact of CVRF on cognitive performance (Launer et al., 2000; Knopman et al., 2001; Korf et al., 2004; Stuerenburg et al., 2005; Irie et al., 2008; Lopez et al., 2008; Erkinjuntti and Gauthier, 2009; Fitzpatrick et al., 2009; Zivadinov et al., 2009; Rasgon et al., 2011; Rouch et al., 2012; Watts et al., 2013). Recent studies have also applied neuroimaging resources in order to show that structural brain changes (e.g., GM loss) are associated to different CVRF (e.g., glycemia level, blood pressure, age, cholesterol levels), separately or in composite measures (Xu et al., 2009; Glodzik et al., 2012).

In the present paper, we aimed to decode cardiovascular risk information from image patterns. In our context, decoding models (or recognition models) related changes in brain pattern (independent variable) to CVRF (dependent variable).

Multivariate approaches can reveal information jointly encoded by all features considered (as a network). Additionally, they can provide predictions in individual level. To the best of our knowledge, this is the first study using PR techniques to investigate the effect of CVRF on the brain based on structural MRI. We hypothesize that brain changes caused by a combination of CVRF risks encompass different brain regions. Thus methods that take into account the joint effect of all voxels in the whole brain can be more sensitive to detect changes than univariate methods.

In the classification of groups categorized according to FCHDR scores, it was possible to discriminate the extreme groups (low *versus* high risk) with statistical significance. However, it should be noted that the indices of diagnostic accuracy obtained were considerably more modest than the accuracy levels found in studies of AD or minor cognitive impairment (Fan et al., 2008; Cui et al., 2011; Davatzikos et al., 2011; Diciotti et al., 2012; Westman et al., 2012). This provides indication that the impact of CVRF in the cognitively preserved elderly population is subtle, even when one applies multivariate statistical approaches of improved sensitivity such as the one employed herein. Regarding the medium group risk, classification in relation to both low and high-risk groups resulted in lower accuracy levels and permutation tests showed these results were not statistically significant. This again underscores the subtlety of the impact of CVRF on brain volumes in cognitively preserved elderly individuals. Medium-risk individuals presented a higher homogeneity in their brain patterns, and this may have prevented their discrimination from the extreme (low and high) risk groups.

This hypothesis is reinforced by the difference in the number of subjects correctly classified in each group in the above mentioned analyses. In high versus medium risk, 62% of the high-risk subjects were correctly classified compared to 43.24% in the medium-risk group. In the same way, in medium versus low risk, 38.10% of the medium-risk subjects were correctly classified and compared to 59.52% in the low-risk group. By correlating FCHDR rates of subjects correctly classified to their respective classification projections, it was possible to observe this inhomogeneity in the asymmetry of projections in Figures 3A,B.

The above findings provide indication that the images from subjects categorized in three groups apparently are not homogeneous enough to discriminate intermediate subjects (medium risk) from low and high-risk subjects. Conversely, given the fact that the correlation between FCHDR rates with their classification projections is high and significant for the three comparisons, our data also indicate that the patterns carry relevant, continuous information regarding the impact of cardiovascular risk on the brain.

Complementary to the classification based on categorized groups, we applied multivariate regression to the complete dataset (without categorization) in order to decode continuous measures from the brain images. This analysis aimed at investigating to what degree brain changes associated to cardiovascular risk are detectable from structural MRI. Using SVR (Support-Vector Regression), parameters were learned from the training subjects’ images. These parameters were used to predict a continuous value for the test subject. Applying SVR in a LOO-CV framework produced a set of predicted values, which was correlated with the set of real values. This correlation was evaluated regarding statistical significance using permutation tests.

Through SVR analysis considering all subjects (both male and female), it was possible to predict the FCHDR score with high statistical significance (*p* < 0.001). This result suggests that anatomical brain changes detectable from MRI images seem to clearly reflect different levels of cardiovascular risk given by the Framingham score. On the other hand, by exploring each risk factor isolated (diabetes, blood pressure, age, total cholesterol, and smoking status), none of them presented significant correlation between real and predicted values. Thus, the SVR analysis allowed us to empirically confirm that brain volume variations predict best the combined influence of different CVRF (as assessed with Framingham scores), rather than by any of the single factors when considered in isolation.

Results of the analyses performed separately in men and women were remarkably different. The correlation between real and predicted FCHDR scores in females was much higher than the correlation obtained when considering the entire population. Additionally, this correlation was not significant when males were considered separately. This is interesting in light of the fact that clinical and epidemiological studies strongly indicate that the prevalence is greater and the outcome is poorer for cardio- and cerebro-vascular disorders in males (Grundy and Jett, 1959; Plavinskaia and Shestov, 1991; Kameneva et al., 2003; Khaksari et al., 2005; Moran et al., 2008). Although gender differences have already been reported in previous studies, the magnitude of these differences in our results suggest that future investigations need to be carried out in order to afford a better understanding of their fundamental basis. However, the gender imbalance in our dataset may contribute for this effect, as men predominate in high-risk group. There was an excess of males from our overall epidemiological cohort that could not be included since previous phases of the investigation due to overt neurological and cerebrovascular-related diseases. Therefore, it is likely that our study design privileged the inclusion of a sub-sample of males with high cardiovascular risk that is less representative of the general elderly population, and this would have exerted an impact on the statistical significance of our findings for males.

When exploring single risk factors in females separately, it was interesting to observe that although two single risk factors (age and smoking status) were significantly predicted, only age remained significant when including the genetic risk factor (the APOE4 allele) as covariate in the model. This finding corroborates once again the importance of considering cardiovascular risks factors jointly when studying their effects on structural brain changes.

Albeit statistically significant, the correlation rates between actual FCHDR scores and values predicted from regression were modest (*r* = 0.2481when considering both male and female together, and *r* = 0.4044 when considering only female subjects). Although in the literature, there are examples where this correlation is very high (e.g., *r* = 0.92 in decoding age from structural MRI from healthy individuals, Franke et al., 2010), there are also examples of applications where the decoding affords low correlations, although still significant (ex. *r* = 0.49 to predict years from onset in Huntington disease, Rizk-Jackson et al., 2011). In our investigation, we are searching for brain alterations due to cardiovascular risk that are presumably subtle, in individuals that are unaffected by neurological or psychiatric disorders and cognitively unimpaired. Although there is evidence in the previous literature suggesting that the individuals with higher FCHDR are more likely to develop AD in the future, it should be noted that at the time the individuals are scanned in our study design, they do not present any symptoms. We believe that the nature of this decoding plays a role in explaining why the correlation between the real and predicted values is not as high as they could be if we were investigating patients actually suffering from dementia.

The results of analyses taking into account the presence of the *APOE* 4 allele suggest the impact of this genetic variation on brain volumes. This is consistent with the findings of a number of previous MRI studies (Yasuda et al., 1998; Hashimoto et al., 2001; Lind et al., 2006; Crivello et al., 2010; Liu et al., 2010; Fennema-Notestine et al., 2011; Lu et al., 2011; Bender and Raz, 2012; Bunce et al., 2012; O’Dwyer et al., 2012; Dowell et al., 2013). It is interesting that the analysis showed different signatures for CVRF-related brain variations in *APOE*-4 allele carriers and non-carriers. A similar difference had already been shown in previous MRI and PET studies that used univariate image analysis methods (Kuller, 2003; Zipser et al., 2007; Deeny et al., 2012; Lo and Jagust, 2012), but this had never been previously demonstrated using the multivariate PR approach as shown herein. Additionally, it is interesting to notice that although analysis in APOE 4 carriers showed higher correlation between real and predicted FCHDR (indicating that the presence of the *APOE* 4 allele modulates the influence of CVRF on regional brain volumes), the correlation obtained from analysis with non-carriers was also significant. This result reinforces the initial hypothesis that CVRF of non-genetic origin also lead to detectable brain changes.

Regarding localization of brain alterations, caution must be exercised when drawing conclusions in regard to the description of the brain regions that contribute to each of the discriminative patterns reported herein. Although the maps enable to visualize the relative relevance of each voxel in predicting the labels, these predictions depend on the whole pattern, so it is not possible to assign results to single brain regions.

In conclusion, our results endorse the suggestion that the severity of the Framingham score for combined cardiovascular risk is related to brain volume changes detectable through global patterns from structural MRI data, when a pattern classification approach is applied. Additionally, these brain changes seem to be actually modulated by the presence of the *APOE* 4 allele but remain detectable when this genetic variable is included in the models as a covariate, suggesting that environmental factors and other genetic influences also play an important role in the emergence of brain changes associated to the degree of cardiovascular risk. The discrimination between individuals with low and high cardiovascular risk hints at a potential clinical usefulness of pattern classification methods to assess the impact of CVRF on the brain on an individual basis. It is important that further studies are conducted to confirm this possibility, given that CVRF are preventable and modifiable, and so are likely to be their effects on the brain.

## Conflict of Interest Statement

The authors declare that the research was conducted in the absence of any commercial or financial relationships that could be construed as a potential conflict of interest.

## Footnotes

^1^Chih-Chung Chang and Chih-Jen Lin, *LIBSVM*: a library for support vector machines, 2001. Software available at http://www.csie.ntu.edu.tw/~cjlin/libsvm/
